# Chang qing formula ameliorates colitis-associated colorectal cancer *via* suppressing IL-17/NF-κB/STAT3 pathway in mice as revealed by network pharmacology study

**DOI:** 10.3389/fphar.2022.893231

**Published:** 2022-08-03

**Authors:** Qihan Luo, Shuo Huang, Lisha Zhao, Jingqun Liu, Qing Ma, Yiheng Wang, Yu Dong, Changyu Li, Ping Qiu

**Affiliations:** ^1^ School of Pharmaceutical Sciences, Zhejiang Chinese Medical University, Hangzhou, China; ^2^ Analytical Testing Center, Zhejiang Academy of Traditional Chinese Medicine, Hangzhou, China; ^3^ School of Basic Medical Science, Zhejiang Chinese Medical University, Hangzhou, China; ^4^ First School of Clinical Medicine, Zhejiang Chinese Medical University, Hangzhou, China

**Keywords:** colitis, colorectal cancer, network pharmacology, IL-17A, MMP9, STAT3

## Abstract

Colitis-associated colorectal cancer (CAC) is a specific type of colorectal cancer (CRC) with high mortality and morbidity, the chronic inflammation in the intestinal mucosal is the characteristic of CAC. Chang Qing formula (CQF) is a Chinese herbal formula used clinically for the treatment of CAC with remarkable clinical efficacy, but its mechanism remains unclear. In the present work, Combined network pharmacology and transcriptomics were used to analyze the potential active ingredients and elucidate molecular mechanism of CQF in treating CAC. Firstly, the constituents migrating to blood of CQF were analyzed and identified by UPLC-Q-TOF-MS/MS, and core genes and pathways were screened by network pharmacology analysis. Encyclopedia of Genes and Genomes (KEGG) analysis showed that the IL-17 signaling pathway involved in CAC may be closely associated with the potential mechanismof action of CQF. Subsequently, the results from animal studies indicated that CQF profoundly reduced tumor numbers and tumor size in AOM/DSS mice. The RNA-seq data was analysed utilizing Ingenuity Pathway Analysis (IPA), and the results supported the idea that CQF exerts a tumour-suppressive effect via the IL-17 signalling pathway. Further studies demonstrated that CQF significantly reduced IL-17A levels, which in turn inhibited NF-κB/IL-6/STAT3 signaling cascade, suppressed MMP9 expression and promoted tumor cell apoptosis. In conclusion, the current study demonstrated that CQF remarkably improved inflammatory tumor microenvironment, and hindered the transformation of inflammation into cancer. These findings may help to design future strategies for the treatment of CAC.

## Introduction

Colorectal cancer (CRC) is a frequently diagnosed cancer with the third-highest cancer morbidity and second-highest cancer mortality rate worldwide (Brody, 2015; Bray et al., 2018). With mortality rates as high as 50%, Colitis-associated CRC (CAC), a subtype of CRC closely related to inflammatory bowel disease (IBD), is more dangerous than sporadic CRC (Feagins et al., 2009). Present in the earliest stage of tumorigenesis, CAC is perhaps one of the typical examples of tumors which closely linked with chronic inflammation. (Lasry et al., 2016). CAC originates from the non-cancerous inflammatory epithelium and evolves into cancer. Inflammation causes severe genotoxic reactions such as DNA damage and mutations to pivotal genes (p53, c-src, k-ras, β-catenin, and APC), leading to the development of CAC in IBD patients (Wu et al., 2018). Surgery followed by chemotherapy is the major strategy for the treatment of CRC (Niu et al., 2015). Adjuvant treatment of cancer patients with traditional Chinese medicine (TCM) has been shown effective in improving patient quality of life and prolonging patient survival (Huang et al., 2020). In China, large numbers of patients with CRC receive adjunctive treatment with TCM (Fang et al., 2012).

Chang Qing formula (CQF) is a TCM used to treat patients with CRC. It is composed of extracts of various plants, including *Cuscuta australis* R. Br (Semen cuscutae), *Epimedium brevicornu* Maxim (Herba epimedii), *Angelica sinensis* (Oliv.)Diels (Radix angelica sinensis), *Pseudostellaria heterophylla* (Miq.) Pax (Radix pseudostellariae), *Taraxacum mongolicum* Hand.-Mazz (Herba taraxaci), *Phellodendron chinense* C.K.Schneid (Cortex phellodendri), *Bupleurum scorzonerifolium* Willd (Radix bupleuri), *Pinellia ternata* (Thunb.) Makino (Rhizoma pinelliae), *Prunus armeniaca* var. *Armeniaca* (Semen armeniacae amarae), and *Glycyrrhiza uralensis* Fisch. ex DC (Radix glycyrrhizae) (Xu et al., 2014; Du et al., 2017). The chemical compounds in these herbs have been revealed to have anti-inflammatory and anti-cancer effects. For example, icariin, a major compound of Herba epimedii, was shown to have wide-ranging anticancer activities *in vitro* and *in vivo* by intervening in the key signaling pathways involved in tumor growth, progression, invasion, and apoptosis (Chen et al., 2016). Several studies have reported that administration of Radix angelica sinensis root extract to mice during early stages of CRC induced by azomethane/dextran sodium sulfate (AOM/DSS) was shown to prevent DNA damage and reduce tumor incidence (Zhao et al., 2017). Herba taraxaci extracts have been reported to have inhibitory effects on breast cancers (Nassan et al., 2018). Besides, Herba taraxaci has been demonstrated to markedly improve IBD by reducing the expression of pro-inflammatory cytokines via a mechanism involving the attenuation of extracellular signal-regulated kinases and suppression of NF-κB and STAT3 (Han et al., 2017). Radix bupleuri is frequently used to treat breast and liver cancer patients in China (Wang et al., 2018), and extracts of Radix bupleuri were found to exert pharmacological activities against cancer and inflammation (Ren et al., 2019). In addition, Cortex phellodendri has been used to treat patients with prostate cancer (Li et al., 2017). Although CQF has been effectively alleviating clinical symptoms and prolonging overall survival of colorectal cancer patients (Xu et al., 2014; Du et al., 2017), its underlying mechanisms of action remain unclear.

**FIGURE 1:**
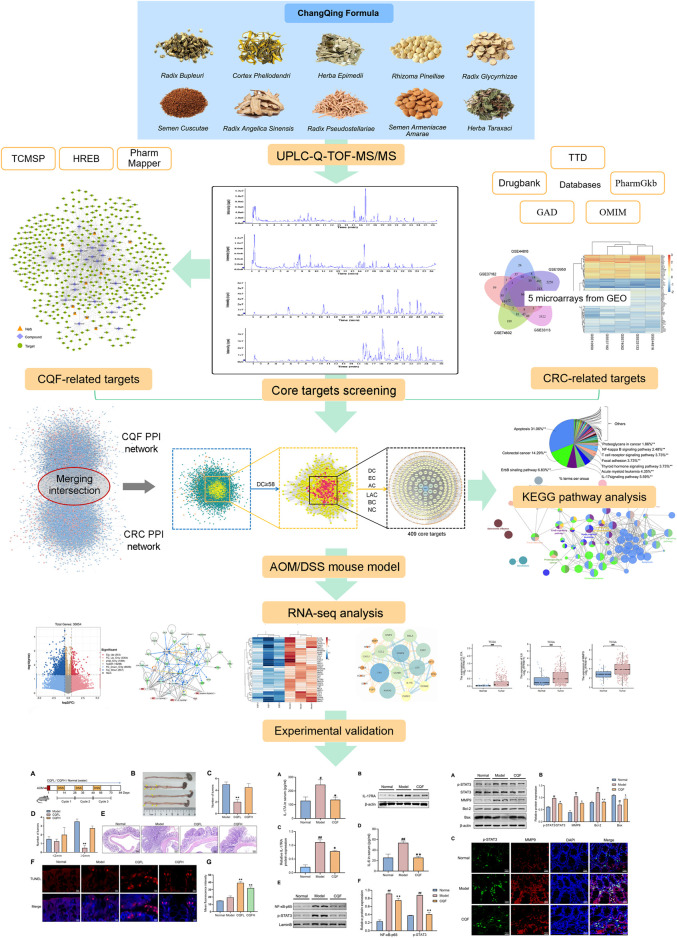
Overall flow of the study, assessing the effects of CQF in the treatment of CRC. All whole components of CQF and the components absorbed into blood were analyzed by UPLC-Q-TOF-MS/MS. Components in blood components were used in network pharmacology to construct the active component-target network diagram screen for core genes affected by CQF in the treatment of CRC. Moreover, components in blood were used in bioinformatics enrichment analysis to identify KEGG signaling pathways affected by CQF. The effects of CQF were subsequently assessed *in vivo* utilizing a mouse model of AOM/DSS-induced CAC.

The present study utilized a combined strategy of serum medicinal chemistry and network pharmacology to identify the active components in CQF, determined their potential molecular targets in CRC, and evaluated the signaling pathways associated with CRC that can be treated by CQF. This study highlighted the holistic and systematic concept of TCM formulas by searching for regulatory mechanisms through network pharmacology and transcriptomic analysis, simplified the complexity of natural compounds in CQF, and provided a reference for further elucidation of the material basis of CQF and its anti-CAC mechanism of action. Figure 1 illustrated the design of this study, which assessed the ability of CQF to treat CAC.

## Materials and methods

### Materials

All the Chinese herbs were provided by Tongde Hospital of Zhejiang Province (Hangzhou, China). All the chemical standards, including l (+)-arginine, proline, adenine, succinic acid, isoleucine, leucine, gallic acid, phenylalanine, protocatechuic acid, l-tryptophan, protocatechuic aldehyde, neo-mangiferin, chlorogenic acid, caffeic acid, amygdalin, mangiferin, rutin, liquiritin, luteoloside, naringin, berberrubine, baicalin, quercetin, epimedin A, epimedin B, epimedin C, icarrin, isorhamnetin, glycyrrhizic acid, limonin, aaikosaponin A, and 3-N-butyl-4,4-dihydrophthalide, were purchased from Shanghai Yuanye Bio-Technology Co. Ltd (Shanghai, China). Azoxymethane (AOM) was obtained from Sigma-Aldrich (St. Louis, MO, United States) and dextran sodium sulfate (DSS) from MP Biomedicals (Santa Ana, CA United States). TUNEL apoptosis assay kits, radioimmunoprecipitation assay (RIPA) buffer, BCA protein assay kits, and anti-MMP9 antibody (AF5234) were purchased from Beyotime (Beijing, China). Mouse IL-6 and IL-17A ELISA kits were from LiankeBio (Hangzhou, China), and antibodies to p-STAT3 (AF3293), STAT3 (AF6294), NF-κB-p65 (BF8005), Bcl-2 (BF9103), Lamin B (AF5161), and β-actin (T0022) were purchased from Affinity Biosciences (Changzhou, China). Antibodies to IL-17RA (sc-376374) and Bax (sc-20067) were purchased from Santa Cruz Biotechnology (Santa Cruz, CA, United States).

### Animals

SPF grade male SD rats weighing 200–220 g and 6-week-old male C57BL/6 mice were provided by Zhejiang Experimental Animal Center (Hangzhou, China) (Certificate No. SCXK (Zhe) 2019–0002). All animals were raised at 22 ± 2°C with relative humidity ranging from 40 to 60%.

### Preparation of CQF decoction

The CQF decoction was prepared by mixing 30 g each of Semen cuscutae, Herba epimedii, Radix angelica sinensis, and Radix pseudostellariae; 21 g of Herba taraxaci; 12 g each of Cortex phellodendri, Radix bupleuri, Rhozoma pinelliae, and Semen armeniacae amarae; 3 g Radix glycyrrhizae, and 2300 ml of pure water. The mixture was heated for 1 h for reflux extraction, followed by filtration. This process was repeated twice to obtain a combined decoction. The final decoction was concentrated using a rotary evaporator, transferred to an evaporating dish and dried to yield 30.4 g extract of CQF, which was stored at −20°C. When the dry extract was administered to rats or mice by gavage, it was reconstituted into a decoction by an appropriate volume of water.

### Preparation of CQF medicated serum

Twenty SD rats were arbitrarily separated into two groups. Rats in the CQF group were orally administered a decoction of CQF (20 g/kg dry extract) twice daily for 5 days, and rats in the control group were administered the same volume of saline, twice daily for 5 days. The rats were fasted for 12 h before being sacrificed. Two hours after the final dose, the rats were anesthetized with sodium pentobarbital (150 mg/kg), and their blood was collected and centrifuged at 3,000 rpm for 15 min at 4 °C to separate serum. The serum samples were stored at −80 °C prior to further use.

### Pretreatment of CQF decoction and serum samples

The CQF decoction was extracted with an equal volume of methanol, vortexed for 1 min, and centrifuged at 14,000 rpm for 20 min at 4°C. The supernatant was loaded into sample vials for UPLC-Q-TOF-MS/MS analysis. Phosphoric acid (60 μl) was added to 3 ml serum, vortexed for 30 s, and ultrasonicated for 1 min. The resulting solution was applied to pre-activated OASIS HLB 3 cc (60 mg) extraction cartridges (Waters, Milford, MA, United States). The cartridges were washed with 3 ml water and 3 ml methanol. The methanol eluates were collected and lyophilized. The residues were dissolved in 100 μl of methanol and centrifuged at 14,000 rpm for 20 min at 4°C, and the supernatant was loaded into sample vials for UPLC-Q-TOF-MS/MS analysis.

### Compound analysis *in Vitro* and *in Vivo*

Compounds were analyzed using the Waters ACQUITY UPLC I-Class PLUS (Waters) and the Sciex X500R QTOF-mass spectrometer system (AB SCIEX, Framingham, MA, United States). The chromatography column was an acquity UPLC BEH C18 (100 × 2.1 mm, 1.7 μm) column (Waters). The mobile phases were acetonitrile (A) and 0.1% formic acid water (B). The gradient elution program consisted of 0–12 min, 99% B-70% B; 12–14 min, 70%–50% B; 14–17 min, 50%–10% B; 17–19 min, 10%–1% B; 19–19.1 min, 1%–99% B; and 19.1–22 min, 99% B. Injection volume was 2.0 μl, the flow rate was 0.3 ml/min, the temperature of the sample tray was 8°C, and the column temperature was maintained at 40°C. Time of flight mass spectrometry was performed with a TurboIonSpray ion source in ESI positive and negative ion scan modes. Secondary mass spectra were acquired by information dependent acquisition (IDA) in high sensitivity mode, with a declustering potential (DP) of ±60 V, and a collision energy of 35 ± 15 EV. IDA was set to exclude isotopes within 4 Da and to monitor 12 candidate ions per cycle 12.

The UPLC-Q-TOF-MS/MS data were collected and processed with SCIEX OS software, which incorporates multiple confidence criteria, including mass accuracy, retention time, isotope, and matching of compound libraries. In the absence of standard products, the target substances were identified using the TCM MS/MS library, a secondary database of TCMs, including more than 1000 TCM compounds, configured by SCIEX OS.

### Prediction of drug targets for CQF

Based on the constituents migrating to blood of CQF detected by UPLC-Q-TOF-MS/MS analysis, the potential targets of bioactive compounds were gathered from the TCMSP (https://old.tcmsp-e.com/tcmsp.php, updated 31 May 2014) (Ru et al., 2014), HERB (http://herb.ac.cn) (Fang et al., 2021), and Pharm Mapper (Liu et al., 2010) (PM, http://www.lilab-ecust.cn/pharmmapper/submitfile.html, updated 10 April 2019) databases. The potential targets of compounds can be directly queried in the TCMSP and HERB databases, whereas the PM data server enables the identification of potential targets of a small molecule probe utilizing a pharmacophore mapping approach. The SDF formats of the 3D structures of these compounds were downloaded from PubChem (Kim et al., 2019) (https://pubchem.ncbi.nlm.nih.gov/, updated 1 January 2019) before submitting them to the PM database to select targets with scores higher than 60 points or the top 50 potential targets.

### Collection of targets related to CRC

The information of CRC-related targets in five databases was obtained by searching the keywords ‘colorectal cancer’. The five databases were the DrugBank database (Wishart et al., 2006) (https://www.drugbank.ca, updated 20 December 2018), the Therapeutic Target Database (Li et al., 2018) (TTD; http://bidd.nus.edu.sg/group/cjttd/, updated 15 September 2017), the Pharmacogenomics Knowledge Implementation (Whirl-Carrillo et al., 2012) (PharmGkb, https://www.pharmgkb.org/, updated 11 January 2019), the Genetic Association Database (Becker et al., 2004) (GAD, http://geneticassociationdb.nih.gov/, updated 1 September 2014), and the Online Mendelian Inheritance in Man database (Amberger and Hamosh, 2017) (OMIM, http://omim.org/, updated 15 January 2019). Differentially expressed genes (DEGs) were collected from five publicly available microarrays (GSE37182, GSE44816, GSE10950, GSE33113, and GSE74602) in the Gene Expression Omnibus database (GEO, https://www.ncbi.nlm.nih.gov/geo/), which recorded information on normal human colon and colon cancer tissue for analysis of DEGs. The DEGs common to the five microarrays were screened by Venn diagram and regarded as CRC-related targets.

### PPI network construction

Protein–protein interactions (PPIs) are essential for maintaining cellular homeostasis and are strongly implicated in disease, suggesting that assessing PPIs can identify drugs of interest (Murakami et al., 2017). The Bisogenet client was designed as a Cytoscape plug-in that enables rapid and easy construction and visualization of biological networks and analyses of graphical topology (Martin et al., 2010). The potential targets screened previously were entered into the Bisogenet to construct PPI networks for CQF and CRC.

### Central network evaluation

The PPI networks of CQF and CRC were merged to obtain the intersection. Six indices were calculated by the plug-in CytoNCA in Cytoscape: ‘betweenness centrality (BC),’ ‘degree centrality (DC),’ ‘eigenvector centrality (EC),’ ‘closeness centrality (CC),’ ‘network centrality (NC),’ and ‘local average connectivity (LAC)’ to analyze the topological properties of each node in the interaction network. To screen for core targets, the candidate genes were selected, with ‘DC’≥2×Median ‘DC’ being the first screening, followed by categorization as ‘DC,’ ‘BC’ ‘EC,’ ‘CC,’ ‘LAC’ and ‘NC’ ≥ 2 × median for secondary screening (Tang et al., 2015).

### KEGG pathway enrichment analysis

ClueGo software is a plug-in of Cytoscape that enriches a large number of candidate targets of pathways or biological functions, with the results visualized as networks (Mlecnik et al., 2018). ClueGo software and the GluePedia plugin were used to perform the Kyoto Encyclopedia of Genes and Genomes (KEGG) enrichment analysis, with *p* ≤ 0.05 and kappa scores ≥0.4 as screening criteria.

### Animals grouping and establishment of AOM/DSS mouse model

The azomethane/dextran sodium sulfate (AOM/DSS) mouse model is an outstanding platform to study the prevention and intervention of CAC. Forty-four C57BL/6 mice were randomly separated into four groups, fourteen in the model (AOM/DSS) group, and ten each in the control group, CQF low-dose (6.25 g/kg dry extract) group and CQF high-dose (25 g/kg dry extract) group. All mice were allowed to acclimate for 1 week. Mice in the CQF groups were gavaged with CQF decoction, and the other two groups were gavaged with the same volume of water. Mice in the AOM/DSS group and the two CQF groups were injected with AOM (10 mg/kg, i. p.) for 1 day after acclimation, whereas mice in the control group were injected with the same volume of normal saline. After 1 week of drinking water, mice were administered 2% DSS in water for another week, replacing the 2% DSS solution every day, followed by tap water for 2 weeks. This process was repeated two more times, with all mice sacrificed at the end of 12 weeks.

### RNA sequencing analysis of AOM/DSS mice

Total RNA from mouse colon tissue was isolated and purified using TRIzol reagent (Invitrogen, Carlsbad, CA, United States) following the manufacturer’s procedure. Poly (A) RNA is purified from 1 μg total RNA using Dynabeads Oligo (dT)25–61,005 (Thermo Fisher, CA, United States) using two rounds of purification. Then the poly(A) RNA was fragmented into small pieces using Magnesium RNA Fragmentation Module (NEB, e6150, United States) under 94°C 5–7 min. Then the cleaved RNA fragments were reverse-transcribed to create the cDNA by SuperScript™ II Reverse Transcriptase (Invitrogen, 1,896,649, United States), which were next used to synthesise U-labeled second-stranded DNAs with *E. coli* DNA polymerase I (NEB, m0209, USA), RNase H (NEB, m0297, United States) and dUTP Solution (Thermo Fisher, R0133, United States). An A-base was then added to the blunt ends of each strand, preparing them for ligation to the indexed adapters. Single- or dual-index adapters were ligated to the fragments, and size selection was performed with AMPureXP beads. After the heat-labile UDG enzyme (NEB, m0280, United States) treatment of the U-labeled second-stranded DNAs, the ligated products were amplified with PCR by the following conditions: initial denaturation at 95°C for 3 min; eight cycles of denaturation at 98°C for 15 s, annealing at 60°C for 15 s, and extension at 72°C for 30 s; and then final extension at 72°C for 5 min. The average insert size for the final cDNA library was 300 ± 50 bp. At last, we performed the 2 × 150 bp paired-end sequencing (PE150) on an illumina Novaseq™ 6,000 (LC-Bio Technology CO. Ltd. Hangzhou, China) following the vendor’s recommended protocol. Transcripts with a *p* value <0.05 and FC > 1.5 were deemed significantly differentially expressed. DEGs in mice were converted to human orthologs for subsequent analysis.

DEGs were uploaded into Ingenuity Pathway Analysis (IPA) software (version 2021, Redwood City, CA, US) for core pathway and network analysis.

Genes derived from the intersection of CQF-related targets and DEGs were input into STRING database (https://cn.string-db.org/) to construct PPI network and visualized by Cytoscape.

### Analysis of data from the cancer genome atlas

RNA-seq data of the human colon cancer (COAD project) were obtained from the TCGA data portal (https://portal.gdc.cancer.gov/), and RNA-seq data, reported as fragments per kilobase per million (FPKM) were log2 transformed.

### Hematoxylin-eosin staining

After sacrifice, the colon tissues of all mice were collected, sectioned longitudinally, fixed in 10% formalin, and embedded in paraffin wax. The colon tissue samples were cut into 4-µm slices, which were stained with hematoxylin and eosin (H&E) and examined under a microscope (Leica, Heidelberg, Germany).

### TUNEL assay

Apoptosis was analyzed using One step TUNEL Apoptosis Assay Kits. Briefly, paraffin sections of colon tissue were dewaxed and rehydrated, followed by the addition of TUNEL detection solution and incubation at 37°C in the dark for 60 min. The sections were incubated with DAPI, and the stained sections were observed and photographed under a fluorescence microscope (Nikon, Tokyo, Japan). The fluorescence intensity of six randomly selected regions in each group was averaged using ImageJ software (Rawak Software Inc. Stuttgart, Germany).

### Enzyme-linked immunosorbent assay

The concentrations of IL-17A and IL-6 in mouse serum samples were analyzed by ELISA according to the instructions provided by the manufacturers of the ELISA assay kits.

### Western blot analysis

Protein samples were extracted from colon tissue using RIPA buffer, which included the protease inhibitor PMSF and a phosphatase inhibitor, according to the manufacturer’s instructions. Protein concentrations were measured with BCA test kits. Proteins from each group were separated on SDS-PAGE gels and transferred to PVDF membranes. The membranes were blocked with 5% nonfat milk at room temperature for 2 h and incubated for 12 h at 4°C with the primary antibodies against IL-17RA (1:100), MMP9 (1:1,000), STAT3 (1:1,000), p-STAT3 (1:1,000), NF-κB-p65 (1:1,000), Bcl-2 (1:1,000), Bax (1:100), β-actin (1:1,000), and LaminB (1:1,000). The membranes were washed three times with TBST prior to incubation with anti-mouse or anti-rabbit secondary antibodies. The bands were scanned by LI-COR Odyssey^®^ Imaging System (LI-COR Biosciences, Lincoln, NE, United States). The gray scale values of the protein bands were calculated using ImageJ software.

### Immunofluorescence assay

Paraffin sections of colon tissue samples were dewaxed, rehydrated, and incubated in 5% nonfat milk for 1 h. The sections were incubated overnight at 4 °C with primary antibodies against p-STAT3 (1:100) and MMP9 (1:100), followed by incubation with FITC-labeled secondary antibodies for 1 h in the dark. Finally the sections were incubated with DAPI, and the stained sections were observed and photographed under a fluorescence microscope (Nikon, Tokyo, Japan).

### Statistical analysis

All data were presented as means ± SEM and compared by one-way analysis of variance (ANOVA) followed by post hoc tests to compare three or more groups, bonferroni correction was applied for multiple comparisons. All statistical analyses were performed utilizing SPSS 22.0 software (SPSS Inc. Chicago, IL, United States), with *p* < 0.05 considered statistically significant.

## Results

### Identification of compounds in CQF decoction and medicated serum

**FIGURE 2:**
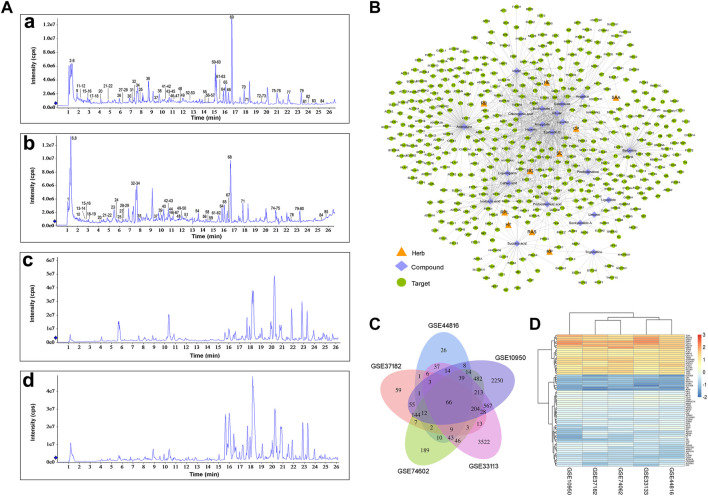
Collection of CQF-related targets and CRC-related targets. **(A)** Total ion chromatographs of CQF samples. a, CQF decoction in ESI^+^ mode; b, CQF decoction in ESI^−^ mode; c, CQF medicated serum in ESI^+^ mode; d, CQF medicated serum in ESI^−^ mode. **(B)** Herb-compound-target network. The network diagram was constructed by connecting Chinese herbs, active compounds, and potential targets. CP: *Cortex phellodendri*; SC: *Semen cuscutae*; RG: *Radix glycyrrhizae*; HT: *Herba taraxaci*; RPL: *Radix pseudostellariae*; RP: *Rhozoma pinelliae*; RAS: *Radix angelica sinensis*; SAA: *Semen armeniacae amarae*; RB: *Radix bupleuri*; HE: *Herba epimedii*. **(C)** Detection of 66 CRC-related targets common to five gene chips. **(D)** Heatmap of the five gene chips.

The chemical composition of CQF decoction and medicated serum were determined by UPLC-Q-TOF-MS/MS analysis, and the compounds absorbed into the blood were clarified in rats. Compounds were matched by molecular mass and fragmentation behaviors using the TCM MS/MS library. These analyses, in both positive and negative ion modes, identified 85 compounds in CQF decoction and 41 compounds in CQF medicated serum sample (Figure 2A). The main constituents migrating to blood were: Trigonelline, Proline, Quinic acid, Stachydrine, Citric acid, Succinic acid, Arbutin, Adenosine, Leucine/Isoleucine, Gallic acid, Phenylalanine, Protocatechuic acid, Salidroside, Hydroxytyrosol, Chlorogenic acid, Caffeic acid, Amygdalin, Mangiferin, Phellodendrine, Isoferulic acid/Ferulic acid, Hyperin, Liquiritin, Isofraxidin, Phloridzin, Liquiritigenin/Isoliquiritigenin, Berberine, Epimedin A, Epimedin B, Epimedin C, Icarrin, Angelicin, Bergapten, Isopimpinellin, Dictamnine, Limonin, Baohuoside I, 3-N-butyl-4,5-dihydrophthalide, Ligustilide, and Corylin. In addition, the mixed standard solution of 32 compounds was analyzed by UPLC-Q-TOF-MS/MS and compared with the label-free identification results to verify the accuracy of the identification method, with the results for all 32 compounds in CQF decoction and 19 compounds in medicated serum being correct. Details of the UPLC-Q-TOF-MS/MS data are shown in Supplementary Table S1.

### Collection of CQF-related targets and CRC-related targets

The compounds in CQF serum with well-defined sources or with high content were chosen to collect targets, ensuring that one to four compounds in each herb were selected. Twenty-two constituents of CQF serum were selected for subsequent analyses: isoferulic acid, phellodendrine, limonin, angelicin, caffeic acid, baohuoside I, protocatechuic acid, ligustilide, icariin, trigonelline, liquiritin, succinic acid, senkyunolide A, hyperin, chlorogenic acid, salidroside, berberine, liquiritigenin, arbutin, epimedin B, amygdalin, and adenosine. A total of 388 potential targets (Supplementary Table S2) were collected from the TCMSP, HERB, and Pharm Mapper databases, and a herb-component-target network was built by Cytoscape (Figure 2B; Supplementary Table S3).

CRC involves the co-regulation of multiple signaling and pathways in the process of tumorigenesis and development. A total of 344 CRC-related targets were collected from five disease databases, with 130, 6, 59, 27, and 163 targets collected from the DrugBank, TTD, PharmGkb, GAD, and OMIM databases, respectively, and the duplicates were removed. In addition, to more comprehensively identify the potential targets of CRC, DEGs were screened in five microarray datasets from the GEO database, using as criteria log2 (|FC|)>1 and *p* < 0.05. Venn diagram analysis identified 66 shared DEGs (Figure 2C). After the integration of all databases, 408 CRC-related targets were collected (Supplementary Table S4).

### PPI network construction and core target screening

**FIGURE 3:**
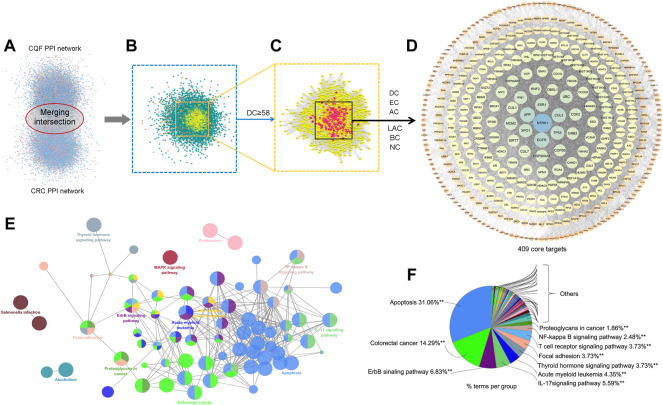
Core target identification and ClueGo pathway analysis. **(A)** PPI networks of CQF-related targets, consisting of 7,700 nodes and 178,773 edges, and CRC-related targets, consisting of 7,343 nodes and 176,169 edges. **(B)** Intersection PPI network, consisting of 5,463 nodes and 147,155 edges. **(C)** Hub PPI network, consisting of 1,307 nodes and 58,424 edges. **(D)** Core-target PPI network, consisting of 409 nodes and 17,708 edges. **(E,F)** A functionally grouped network of categories enriched for the target genes. GO terms are represented as nodes, and node size represents the significance of term enrichment. Functionally related groups partially overlapped. Only the most significant term in the group was labeled. Representative enriched pathway (*p* < 0.05) interactions among the main CQF targets.

To explore the potential core targets of CQF in the treatment of CRC, a CQF-related PPI network, consisting of 7,700 nodes and 178,773 edges, and a CRC-related PPI network, consisting of 7,343 nodes and 176,169 edges, were constructed using Bisogenet (Figure 3A). A merger of two networks showed that the intersection of the PPI networks consisted of 5,463 nodes and 147,155 edges (Figure 3B). The topologic features of the network were calculated and the hub network further screened utilizing CytoNCA. Primary screening was performed by the criterion of ‘DC’ ≥ 58 (Figure 3C), with the hub network further screened by the criteria of ‘DC’ ≥ 107, ‘EC’ ≥ 0.01667164, ‘LAC’ ≥ 15.98717949, ‘BC’ ≥ 530.053963, ‘CC’ ≥ 0.502887948 and ‘NC’ ≥ 20.394804. These analyses resulted in the construction of a central PPI network (CPPI) consisting of 409 core candidate targets, which may play important roles in the treatment of CRC. (Figure 3D). The topological characteristics of all of these targets were shown in Supplementary Table S5, the high-definition big figures of the networks were presented in Supplementary Figures S1–S4.

### KEGG pathway enrichment analysis

To further assess the mechanism of CQF in the treatment of CRC, the 409 core targets were input into ClueGo for KEGG pathway enrichment analysis. All core targets were detected in 41 KEGG pathways (Supplementary Table S6), with the main pathways shown in Figures 3E,F. Of these 41 pathways, the apoptosis, ErbB signaling, and IL-17 signaling pathways showed higher priority, suggesting that they might play a role in the treatment of CRC by CQF. IL-17 (IL-17A) was a crucial inflammatory cytokine shown to promote colonic inflammation and CRC tumorigenesis, especially the development of CAC (Ning et al., 2015; Xie et al., 2015).

### Inhibitory effect of CQF on colitis-associated cancer in AOM/DSS-induced mice

**FIGURE 4:**
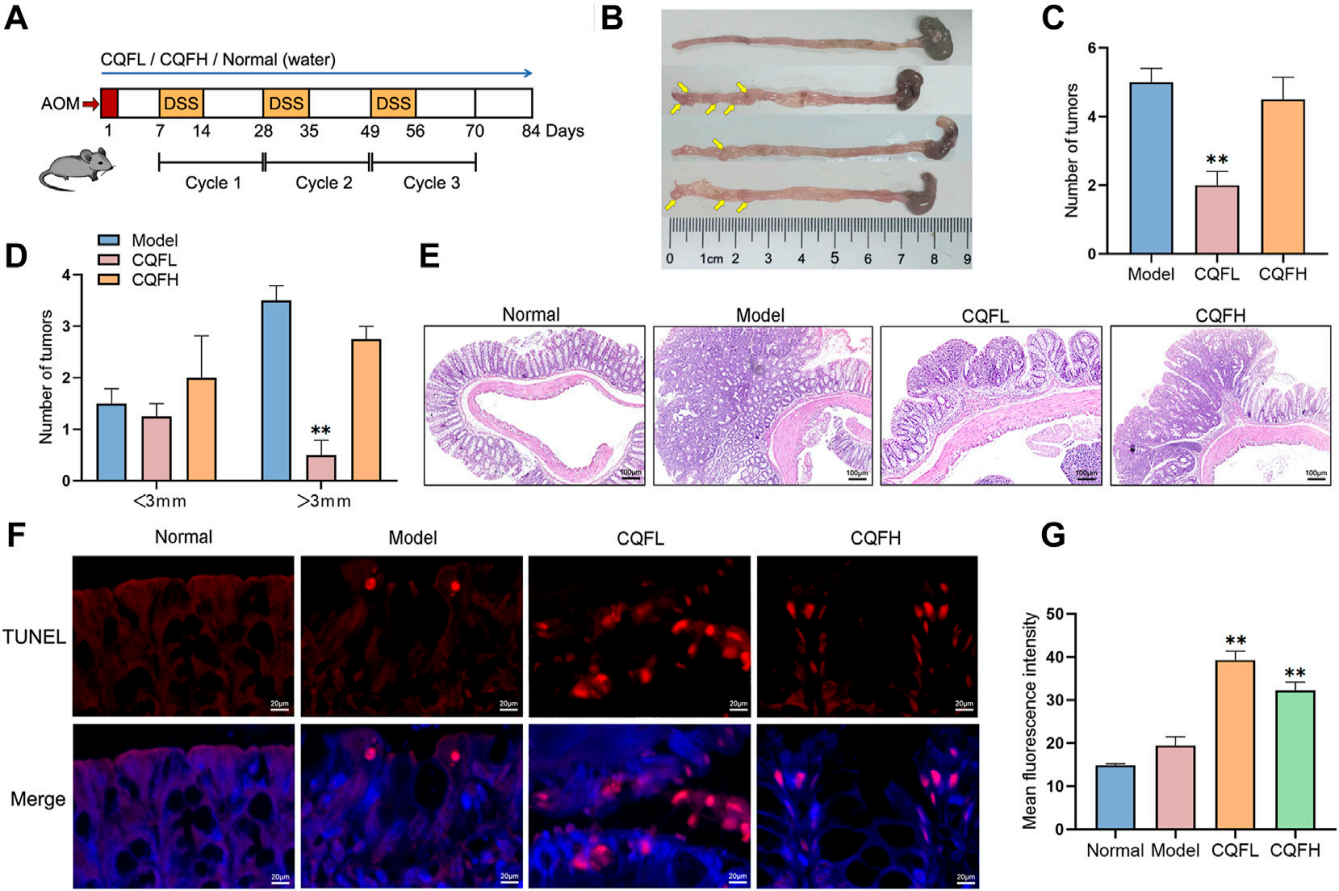
Effect of CQF on colitis-associated cancer in AOM/DSS-treated mice. **(A)** Schematic diagram of the AOM/DSS model. **(B)** Photographs of mouse colon tissues, with tumors indicated by yellow arrows. **(C,D)** Number and size of colorectal tumors in mice (n = 6 each). **(E)** H&E staining of colon tissues. **(F)** TUNEL staining of colon tissues. **(G)** Mean fluorescence intensity, calculated from six randomly selected localized areas in each group. Results are expressed as mean ± SEM. ^*^ *p* < 0.05*,* ^**^ *p* < 0.01*,* compared with AOM/DSS-treated mice. ^*###*^ *p <* 0.01, compared with the normal group.

To verify the antitumor mechanism of CQF, a CAC mouse model was constructed by exposing to AOM and DSS (Figure 4A). The general symptoms of CAC were observed in these mice, including stool irregularity, hematochezia, and shortening of the colorectum, indicating that AOM/DSS induced severe colonic inflammation. However, administration of CQF to these mice significantly inhibited the shortening of the colorectum (Figure 4B), as well as significantly reducing the number and volume of colorectal tumors, with tumors of diameter >3 mm being extremely rare in mice treated with 6.25 g/kg CQF (Figures 4C,D). To confirm the inhibitory effect of CQF on tumors, colorectal tissue samples were examined histologically. Examination of samples from normal mice showed that their colon glands were arranged in an orderly and compact manner without inflammatory damage, whereas Sections from mice treated with AOM/DSS showed atypical hyperplasia, deformed glands, and disordered arrangement and surface erosion. Treatment of AOM/DSS-induced mice with CQF reduced these pathological symptoms, indicating that CQF had an inhibitory effect on colorectal tumors, especially in those treated with 6.25 g/kg CQF (Figure 4E).

The levels of apoptosis in these mice were assessed by TUNEL staining analysis of these colorectal tissue samples. Samples from normal mice and AOM/DSS-treated mice showed few positive cells, whereas samples from AOM/DSS-induced mice treated with 6.25 g/kg CQF showed large numbers of positive cells (Figure 4F). Calculation of the mean fluorescence intensity using ImageJ software showed that the mean fluorescence intensity was significantly higher in CQF groups than in model group, suggesting that CQF promoted the apoptosis of cells in colorectal lesions, thereby inhibiting CRC development (Figure 4G). Taken together, these findings showed that 6.25 g/kg CQF had a greater inhibitory effect on AOM/DSS-induced CAC than 25 g/kg CQF. Therefore, in subsequent experiments, the effects of the lower concentration of CQF were further analyzed.

### Transcriptomic analysis results of CQF-treated AOM/DSS-induced mice

**FIGURE 5:**
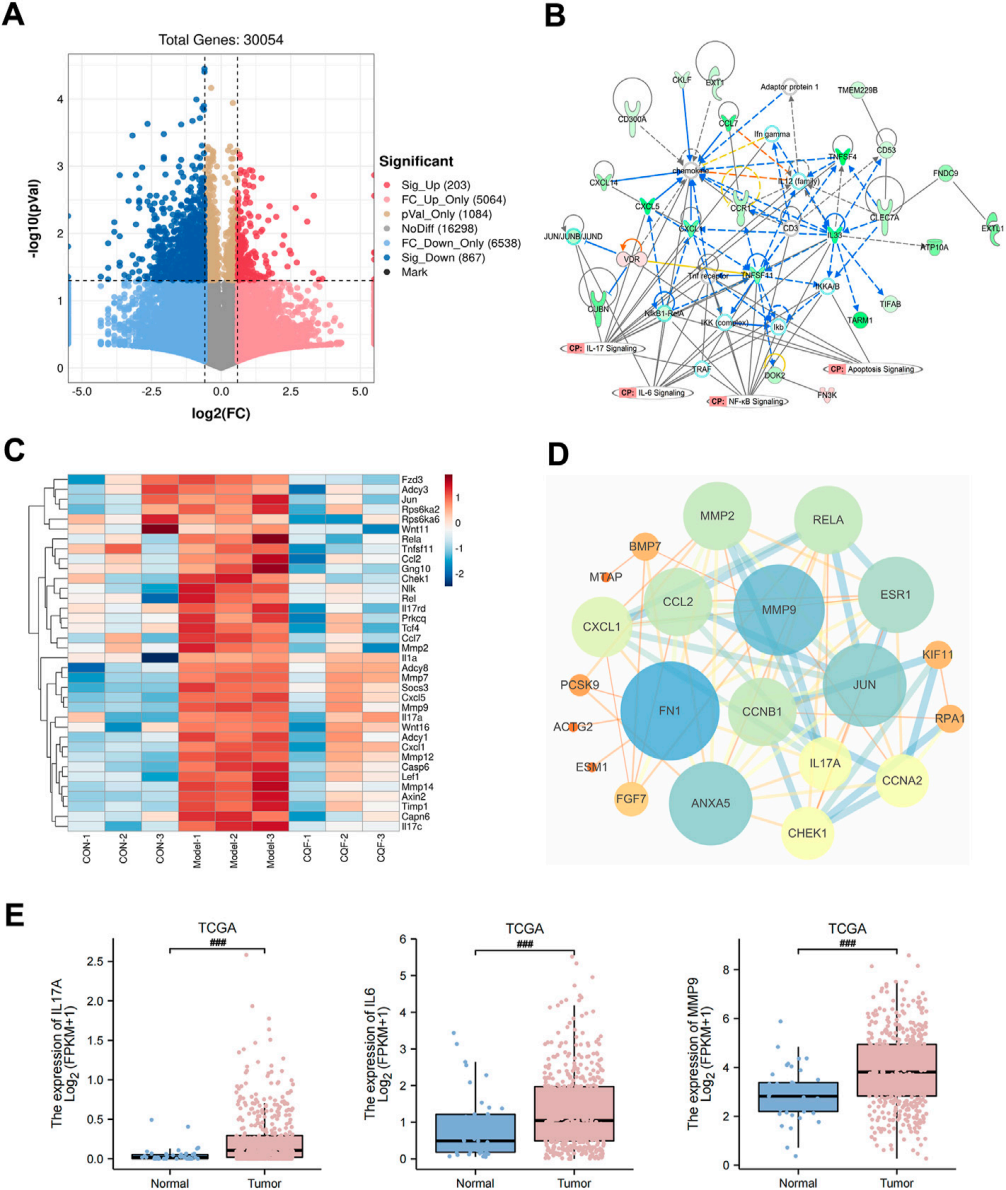
RNA-seq analysis of CQF-treated AOM/DSS-induced CAC mice. **(A)** Volcano plot of DEGs between model group and CQF group. Transcripts with a *p* value <0.05 and FC > 1.5 were deemed significantly differentially expressed. **(B)** IPA enrichment analysis of DEGs. **(C)** Heatmap of genes with functions related to IL-17 pathway, IL-6 pathway, apoptosis, and colorectal cancer metastasis among the DEGs. **(D)** PPI network of the intersection of CQF-related targets and DEGs. **(E)** TCGA showed increased transcript levels of IL-17A, IL-6, MMP9 in human colon cancer samples.

The RNA sequencing was performed on mouse colon tissues, and transcripts with a *p* value <0.05 and FC > 1.5 were considered to be significantly differentially expressed between the model group and the CQF group (6.25 g/kg). The volcano plot was shown in Figure 5A, and compared with the AOM/DSS model group, a total of 1,070 DEGs were generated after CQF treatment, including 203 upregulated genes and 867 downregulated genes. These 1,070 DEGs were input into IPA software for core network analysis and enriched to four vital pathways: IL-17 pathway, IL-6 pathway, NF- κB pathway, and apoptosis pathway (Figure 5B). Subsequently, the genes related to the IL-17 pathway, IL-6 pathway, apoptosis, and colorectal cancer metastasis in DEGs were extracted and plotted with a heat map (Figure 5C), which showed that CQF restricted the transcript levels of genes associated with inflammation (*Il17A*, *Jun*, *Mmp9*, *Rela*, *Ccl2*, *Cxcl1*, etc.), apoptosis (*Rela*, *Fgfr1*, *Il1a*, *Chek1*, *Casp6*, etc.) and colorectal cancer metastasis (*Mmp7*, *Mmp9*, *Lef1*, *Axin2*, *Wnt11*, *Tcf4*, *Mmp12*, *Mmp14*, *Mmp2*, etc.). In addition, a PPI network was constructed with the intersection genes of CQF-related targets and DEGs, and the targets with a high degree were FN1, MMP9, JUN, RELA, CCL2, CXCL1, IL17A, CHEK1, MMP2, and so on (Figure 5D). The data of some key molecules associated with the development of CAC were collected from the TCGA database, which showed that mRNA levels of IL-17A, IL-6, and MMP9 were significantly enhanced in CRC samples than in normal colon tissues (Figure 5E), indicating that the increased expression of inflammatory cytokines and MMP9 were involved in the development and progression of colorectal cancer. Taken together, CQF was suggested to attenuated the activation of the IL-17 signaling pathway and suppress the invasion and metastasis of colorectal cancer.

### CQF decreased the levels of IL-17A and impeded the activation of NF-kb/IL-6/STAT3 signaling cascade

**FIGURE 6:**
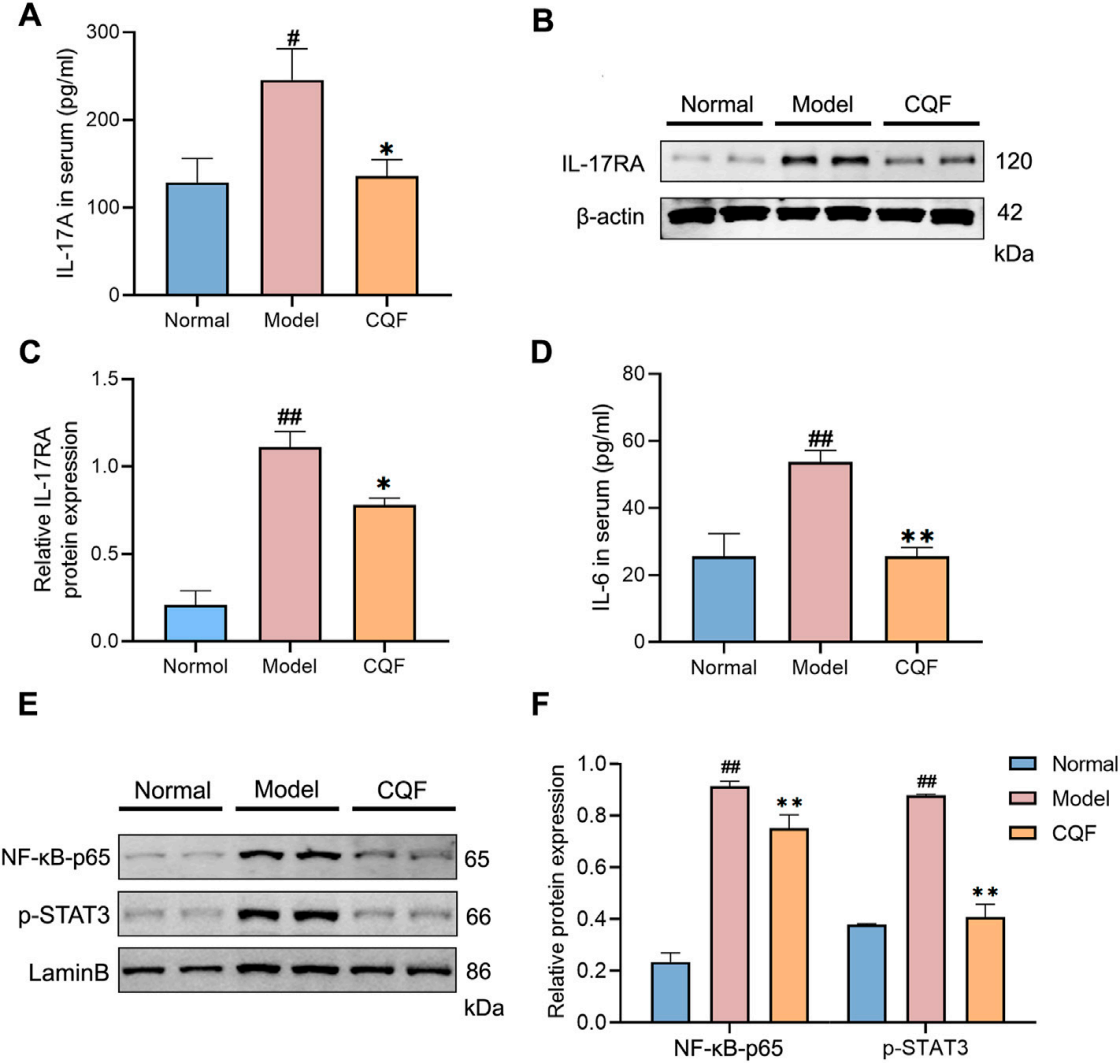
CQF decreased the level of IL-17A and impeded the activation of NF-kB/IL-6/STAT3 signaling cascade. **(A)** IL-17A concentrations in serum samples. **(B–C)** Expression of IL-17RA protein in colon tissue samples (n = 4 per group). **(D)** IL-6 concentrations in serum samples (n = 6 per group). **(E,F)** Nuclear expression of NF-κB-p65 and p-STAT3 proteins in colon tissue samples (n = 4 per group). Results are expressed as mean ± SEM. ^*#*^ *p <* 0.05, ^*##*^ *p <* 0.01, compared with normal mice; ^*^ *p <* 0.05, ^**^ *p <* 0.01, compared with AOM/DSS-treated mice.

To further evaluate the regulation of CQF on the IL-17 signaling pathway, key cytokines and protein expression were detected. IL-17A is a cytokine secreted by Th17 cells and acts by binding to its type A receptor (IL17-RA). Compared with normal mice, mice treated with AOM/DSS showed significantly higher serum levels of IL-17A and tissue expression of IL-17RA, with CQF significantly inhibiting the increases (Figures 6A–C). Moreover, compared with normal mice, mice treated with AOM/DSS showed increases in IL-6 levels as well as nuclear expression of NF-kB-p65 and p-STAT3, with all of these increases being markedly down-regulated after CQF treatment (Figures 6D–F). Taken together, these findings indicated that CQF inhibited the elevation of IL-17A and impeded the activation of the downstream NF-kB/IL-6/STAT3 signaling cascade.

### CQF suppressed the phosphorylation of STAT3 and altered the expression of its downstream proteins

**FIGURE 7:**
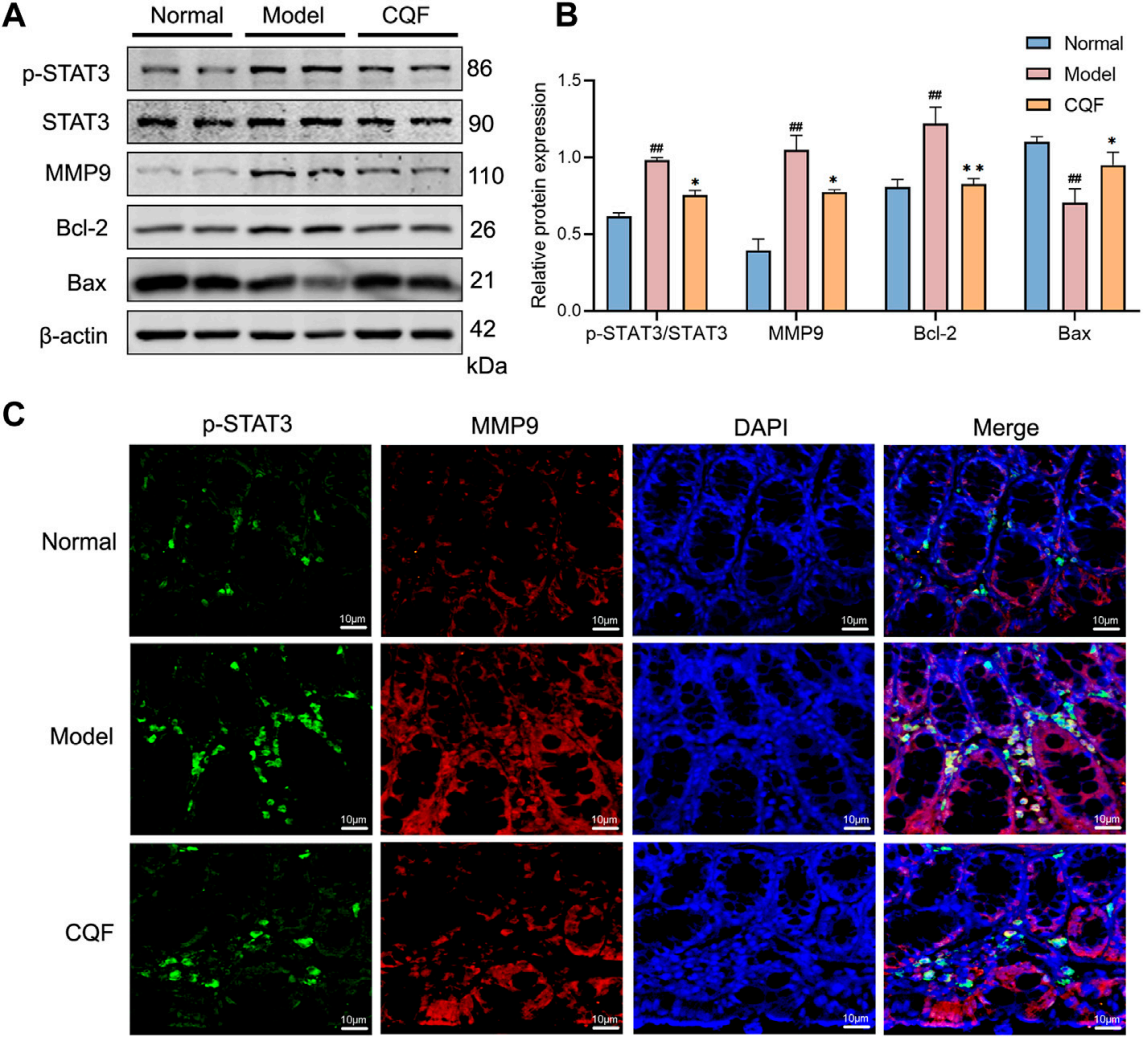
CQF suppressed the phosphorylation of STAT3 and altered the expression of its downstream proteins. **(A,B)** Expression of MMP9, p-STAT3, STAT3, Bcl-2, and Bax proteins in colon tissue (n = 4 per group). **(C)** Immunofluorescence staining of colon tissue, green: p-STAT3, red: MMP9, blue: DAPI. Values are expressed as mean ± SEM. ^*#*^ *p < 0.05,* ^*##*^ *p <* 0.01, compared with normal mice; ^*^ *p <* 0.05, ^**^ *p <* 0.01, compared with AOM/DSS-treated mice.

The phosphorylation of STAT3 may play an essential part in regulating cancer cell growth and proliferation. Treatment with AOM/DSS significantly activated STAT3, leading to the up-regulation of MMP9 and Bcl-2 and the down-regulation of Bax. However, CQF treatment of these mice markedly suppressed the phosphorylation of STAT3, as well as decreased the expression of MMP9 and promoted apoptosis. (Figures 7A,B). Immunofluorescence results showed that, compared with normal mice, treatment with AOM/DSS enhanced the numbers of cells simultaneously expressing p-STAT3 and MMP9, whereas treatment of AOM/DSS-induced mice with CQF reduced the numbers of doubly positive cells when compared with the model group (Figure 7C). These findings implied that suppression of STAT3 phosphorylation by CQF might play a significant role in preventing the development of CAC.

## Discussion

**FIGURE 8:**
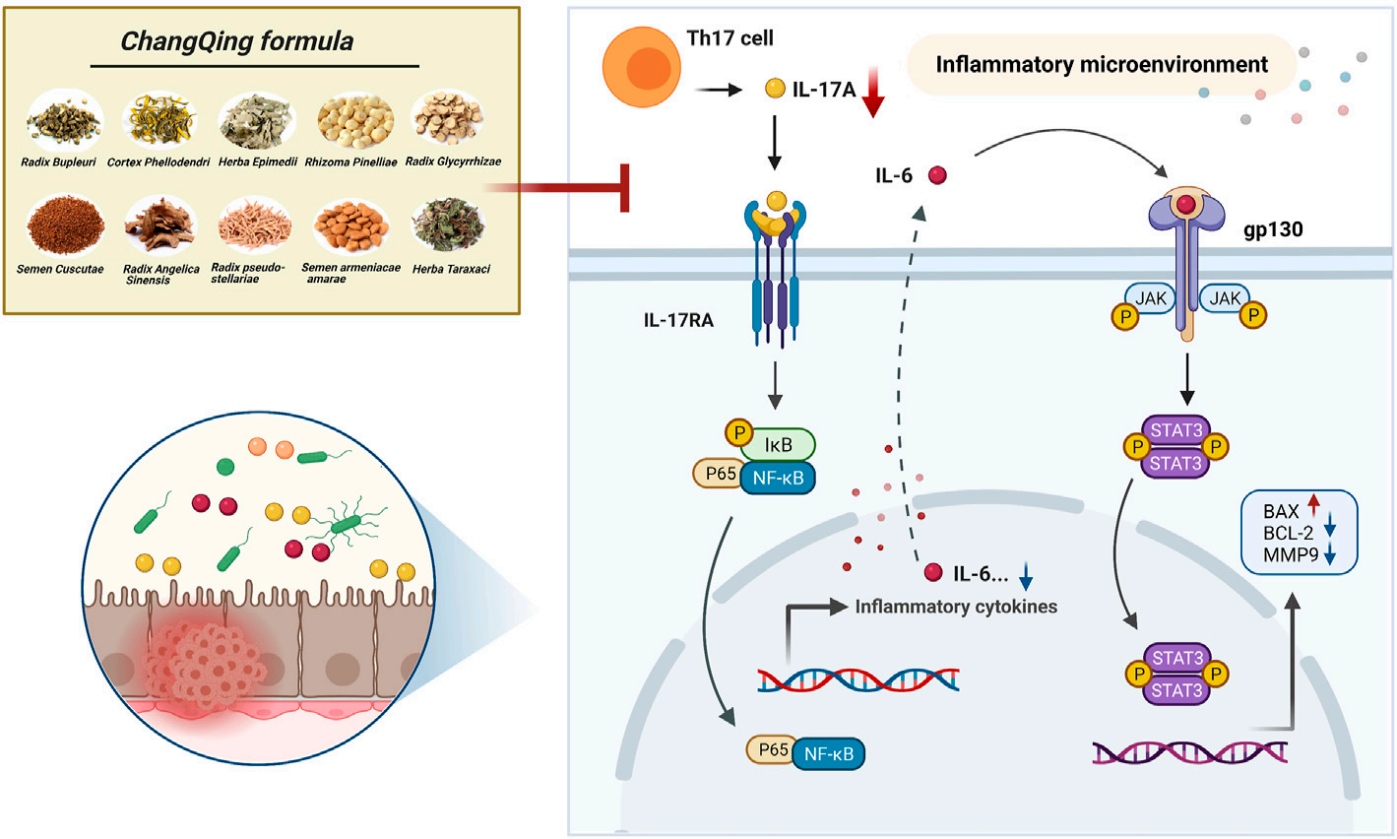
Diagram showing a hypothesized mechanism by which Chang Qing formula is effective in the treatment of colitis-associated colon cancer.

Although TCMs have therapeutic properties, they are composed of multiple components, thus making it difficult to determine their therapeutic mechanisms of action. Serum medicinal chemistry regards constituents migrating to blood and their metabolites as being active compounds with medicinal effects, suggesting that analysis of blood components could determine the mechanisms of action of TCMs (Li et al., 2016; Wang et al., 2021). In the present study, 409 potential core targets of CQF for the treatment of CRC were screened utilizing network pharmacology based on constituents migrating to blood. Multiple pathways were found to be involved, including the apoptosis, ErbB signaling pathway, IL-17 signaling pathway, and NF-κB signaling pathway. Subsequently, an AOM/DSS-induced CAC mouse model was established and treated with CQF decoction. *In vivo* experiments indicate that CQF profoundly reduced tumor numbers and tumor size in AOM/DSS mice. Subsequently, transcriptomic analysis were futher performed to explore potential key genes regulated by CQF. Finally, the ability of CQF to inhibit the levels of IL-17A and the downstream NF-κB/IL-6/STAT3 signaling cascade was verified by a series of molecular biological experiments. This study suggested a novel approach to systematically elucidate the mechanisms of action of the TCM herbal formula CQF. The hypothesized mechanism of CQF in treating CAC is shown in Figure 8.

Chronic inflammation is one of the characteristics of tumors and many cancers develop in response to prolonged inflammation, CAC was the most highly represented cancer type (Tan et al., 2020). The incidence of CRC has been reported to be 60% higher in patients with IBD, particularly those with ulcerative colitis (UC), than in the general population (Friis et al., 2015). Inflammatory cytokines play important roles in tumor development (Zou et al., 2020), the persistent inflammation can activate the proliferation of premalignant cells and inhibit their apoptosis (Wang and Karin, 2015). Noteworthy, CAC can be delayed or even prevented by treatment with anti-inflammatory drugs, suggesting that inflammatory processes are involved in tumor onset (Lasry et al., 2016). IL-17A, an inflammatory mediator mainly secreted by Th17 cells, has been shown to promote tumor development and play a strong proinflammatory role in immune diseases (McGeachy et al., 2019). IL-17A binds to its type A receptor (IL-17RA), with several studies showing that IL-17A and IL-17RA expression is higher in colon tumor than in normal colon tissues (Wang et al., 2014; Xie et al., 2015; Wu et al., 2019). IL-17A was shown to activate NF-κB and induce NF-κB-dependent cytokine production, followed by the induction of proinflammatory cytokines, chemokines, antimicrobial peptides (AMPS), matrix metalloproteinases (MMPs), and inflammatory effectors. Moreover, IL-17A can synergize with many other inflammatory stimuli, further enhancing its activity and promoting the transition of inflammation to cancer (Amatya et al., 2017). In the present study, KEGG pathway analysis of network pharmacology and IPA analysis of transcriptomics indicated that IL-17 signaling pathway was significantly enriched, and the study further validated the involvement of the IL-17 signaling pathway in CAC development. Treatment of mice with AOM/DSS significantly increased the levels of expression of IL-17A and IL-17RA in serum and colon tissues, respectively, findings consistent with those of previous reports. This study found that treatment of these mice with CQF reduced the expression of both IL-17A and IL-17RA, suggesting that the therapeutic effect of CQF in CAC may be due to its downregulation of the IL-17 signaling pathway.

According to the results of RNA-seq, the genes related to inflammation, apoptosis, and metastasis in DEGs were extracted, combining the intersection genes of CQF-related targets with DEGs, we identified several important potential targets downstream of IL17A, they were IL6, RELA (NF-κB p65), and MMP9. NF-κB and STAT3 signalings in lamina propria (LP) immune cells and intestinal epithelial cells (IECs) were shown to play a crucial role in the initiation and promotion of CAC (Zhang et al., 2021). NF-κB expression was reported to be abnormally elevated in more than 50% of CRCs and colitis-associated tumors (Karin, 2006). Upon IL-17A stimulation, NF-κB dimers (p65 and p50) enter the nucleus, where they become constitutively activated and further upregulate major inflammatory factors, such as TNFα, IL-6, IL-1, and IL-8 (Fan et al., 2013). Our study found that CQF inhibited the AOM/DSS-induced elevated nuclear expression of NF-κB and p-STAT3 as well as increased levels of IL-6 in serum. The NF-κB/IL-6/STAT3 signaling cascade was closely associated with the inflammatory response, which induced proliferation and remodeling of epithelial cells and then promoted tumor development (Grivennikov et al., 2009). IL-6 binding to the receptor gp130 drived STAT3 activation in IECs (Sharma et al., 2022), leading to transcriptional expression of downstream target genes, which in turn promote cell survival and proliferation (De Simone et al., 2013). In addition, activated STAT3 prolonged NF-κB nuclear retention, with their synergistic activity maintaining and exacerbating inflammatory responses (De Simone et al., 2015). Although STAT3 activation was critical for the immune function of IECs, exacerbated immune responses lead to epithelial barrier dysfunction, which further promoted inflammatory cell infiltration and aggravated tissue damage in the intestinal mucosa. These findings suggested that CAC results from persistent inflammation in the intestinal mucosa, occurring in an “inflammation-dysplasia-cancer” sequence (Gui et al., 2020; Bocchetti et al., 2021), with the NF-κB/IL-6/STAT3 signaling cascade being essential for maintaining the immune microenvironment and driving the transition from inflammation to cancer. Furthermore, pharmacological experiments showed that AOM/DSS activated STAT3 and enhanced the expression of the downstream genes Bcl-2 and MMP9 while decreasing Bax expression, with these abnormal levels of expression being inhibited by CQF treatment. It is well known that a decreased Bax/Bcl-2 ratio indicated that apoptosis was inhibited, and MMP9 was an important enzyme that degraded the extracellular matrix (ECM) during tumor metastasis and was highly associated with tumor invasion (Ha et al., 2019; Buttacavoli et al., 2021; Idiiatullina et al., 2021).

Moreover, the results of RNA-seq implicated other key genes that can also serve as strong evidence that CQF inhibits development of CAC. For example, chemokine ligand CCL2 and CXCL1 were both genes related to IL-17 pathway, CCL2 promoted cancer cell migration and recruited immunosuppressive cells to the tumor microenvironment, favoring cancer development (Xu et al., 2021), CXCL1 was associated with immunosuppression and tumorigenesis in various tumor types, blocking CXCL1 in mice can suppress CAC progression (Liu et al., 2019). Several lines of evidence suggest that CHEK1 responded to DNA damage by initiating cell cycle arrest, thus providing time for cells to repair damage and escape cell withering before restoring cell cycle, which has prognostic significance in human colorectal cancer (Gali-Muhtasib et al., 2008). Futhermore, FN1 was critical for regulating tumor metastasis and invasion, with its protein being a marker of epithelial mesenchymal transition (EMT) (Xie et al., 2019).

## Conclusion

In summary, this study systematically indicated that CQF can exert a significant inhibitory effect on CAC development, as well as revealing the underlying mechanisms of action of CQF through a combination of serum medicinal chemistry, network pharmacology and transcriptomics analyses. Taken together, these findings suggested that CQF can ameliorate CAC in mice by reducing the levels of expression of IL-17A and NF-κB and by regulating the inflammatory tumor microenvironment, subsequently suppressing the IL-6/STAT3 signaling pathway that was involved in tumor angiogenesis and invasiveness. The present study provided novel insights into the role of TCM in the treatment of CAC, as well as supporting future drug development from natural products.

## Data Availability

The datasets presented in this study can be found in online repositories. The names of the repository/repositories and accession number(s) can be found in the article/Supplementary Material.

## Glossary

ANOVA: analysis of variance
AOM: azomethane
Bax: BCL2-Associated X
BC: betweenness centrality
Bcl-2: B-cell lymphoma-2
CAC: colitis-associated colorectal cancer
CC: closeness centrality
CQF: chang qing formula
CRC: colorectal cancer
DAPI: 4′,6-diamidino-2-phenylindole
DC: degree centrality
DEG: differentially expressed gene
DSS: dextran sodium sulfate
EC: eigenvector centrality
ECM: extracellular matrix
ELISA: enzyme-linked immunosorbent assay
EMT: epithelial mesenchymal transition
GAD: genetic association database
GEO: gene expression omnibus database
H&E: hematoxylin and eosin
IBD: inflammatory bowel disease
IDA: information dependent acquisition
IL-17A: interleukin-17A
IL-17RA: interleukin-17 receptor A
IL-6: interleukin-6
IPA: ingenuity pathway analysis
KEGG: encyclopedia of genes and genomes
LAC: local average connectivity
MMP9: matrix metalloproteinase-9
NC: network centrality
NF-κB: nuclear factor kappa-B
OMIM: online mendelian inheritance in man database
PM: pharm mapper
PPI: Protein–protein interaction
RNA-seq: RNA sequencing
STAT3: signal transducer and activator of transcription three
TCGA: the cancer genome atlas
TCM,: traditional chinese medicine
TCMSP: traditional chinese medicine systems pharmacology database and analysis platform
TTD: therapeutic target database
TUNEL: TdT-mediated dUTP nick-end labeling
UPLC-Q-TOF-MS/MS: ultra performance liquid chromatography—quadrupole—time of flight-mass spectrometry
