# Supplementary material for: Chang qing formula ameliorates colitis-associated colorectal cancer via suppressing IL-17/NF-κB/STAT3 pathway in mice as revealed by network pharmacology study
Source: Front Pharmacol. 2022 Aug 3;13:893231. doi: 10.3389/fphar.2022.893231 (PMC9382085; doi:10.3389/fphar.2022.893231)
Supplement: Supplementary file 4 [file DataSheet1.pdf]

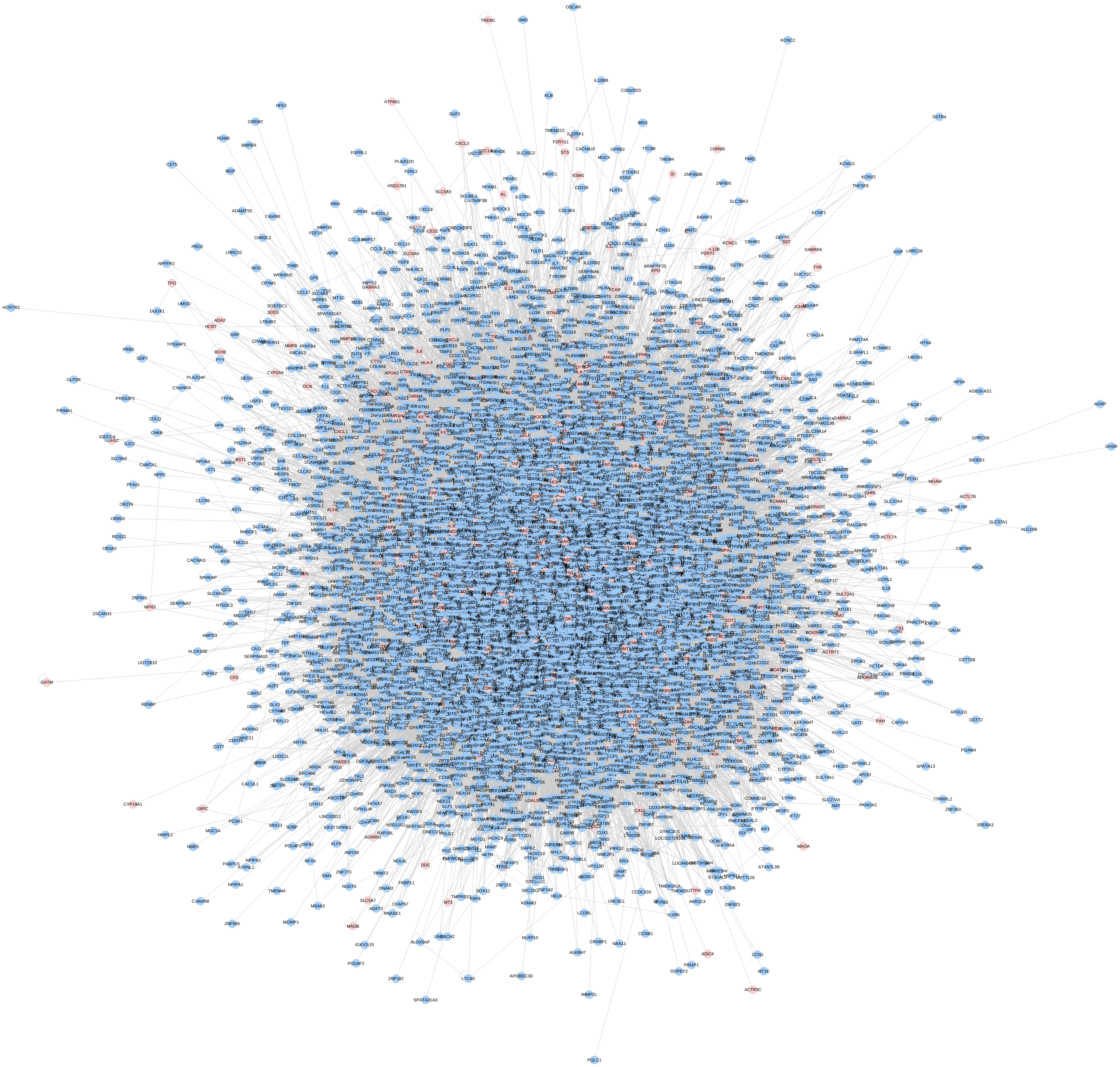

Supplementary Figure 1: PPI network of CQF-related targets. 7700 nodes and 178,773 edges were identified.

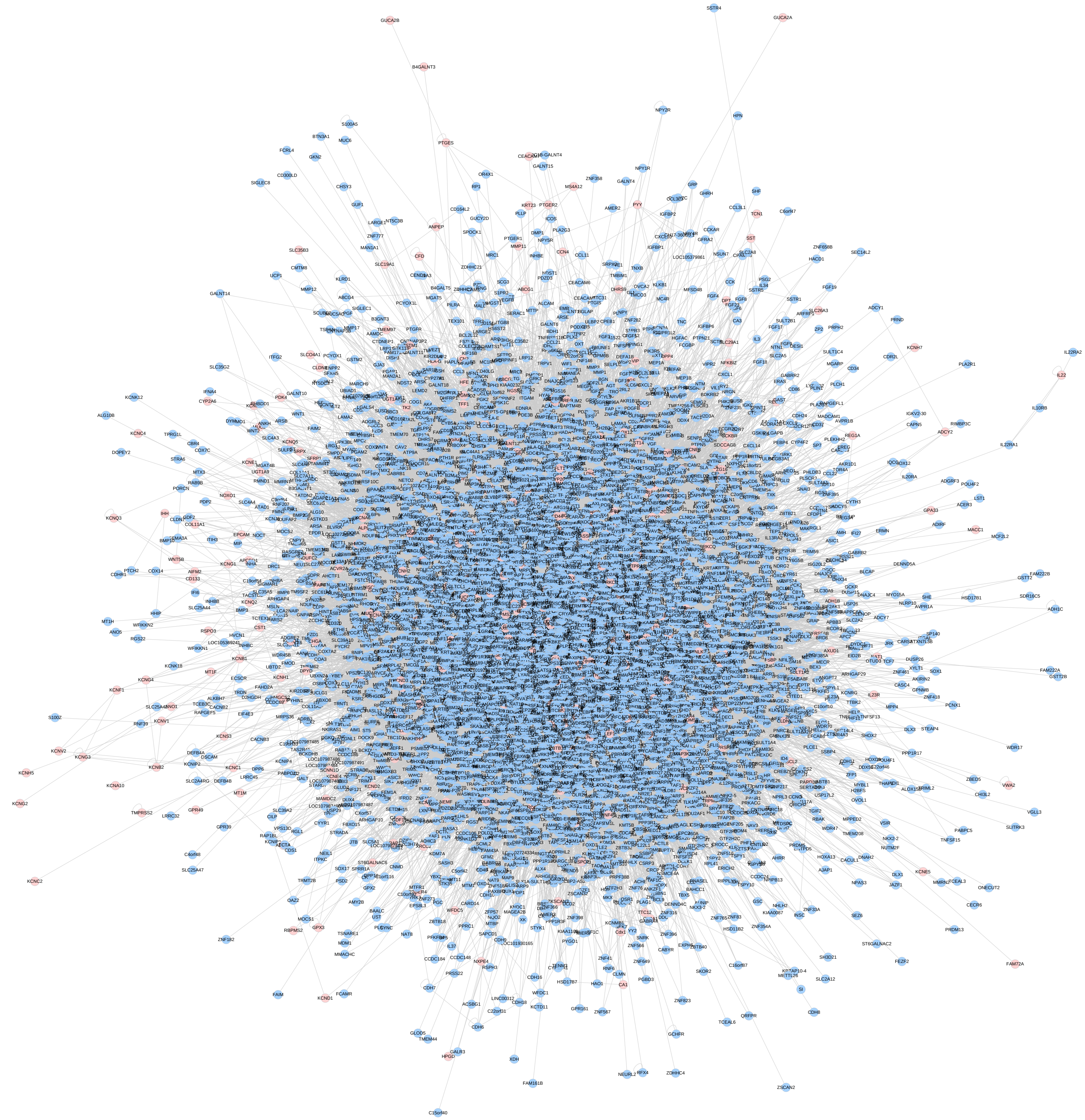

Supplementary Figure 2: PPI network of CRC-related targets. 7343 nodes and 176,169 edges were identified.

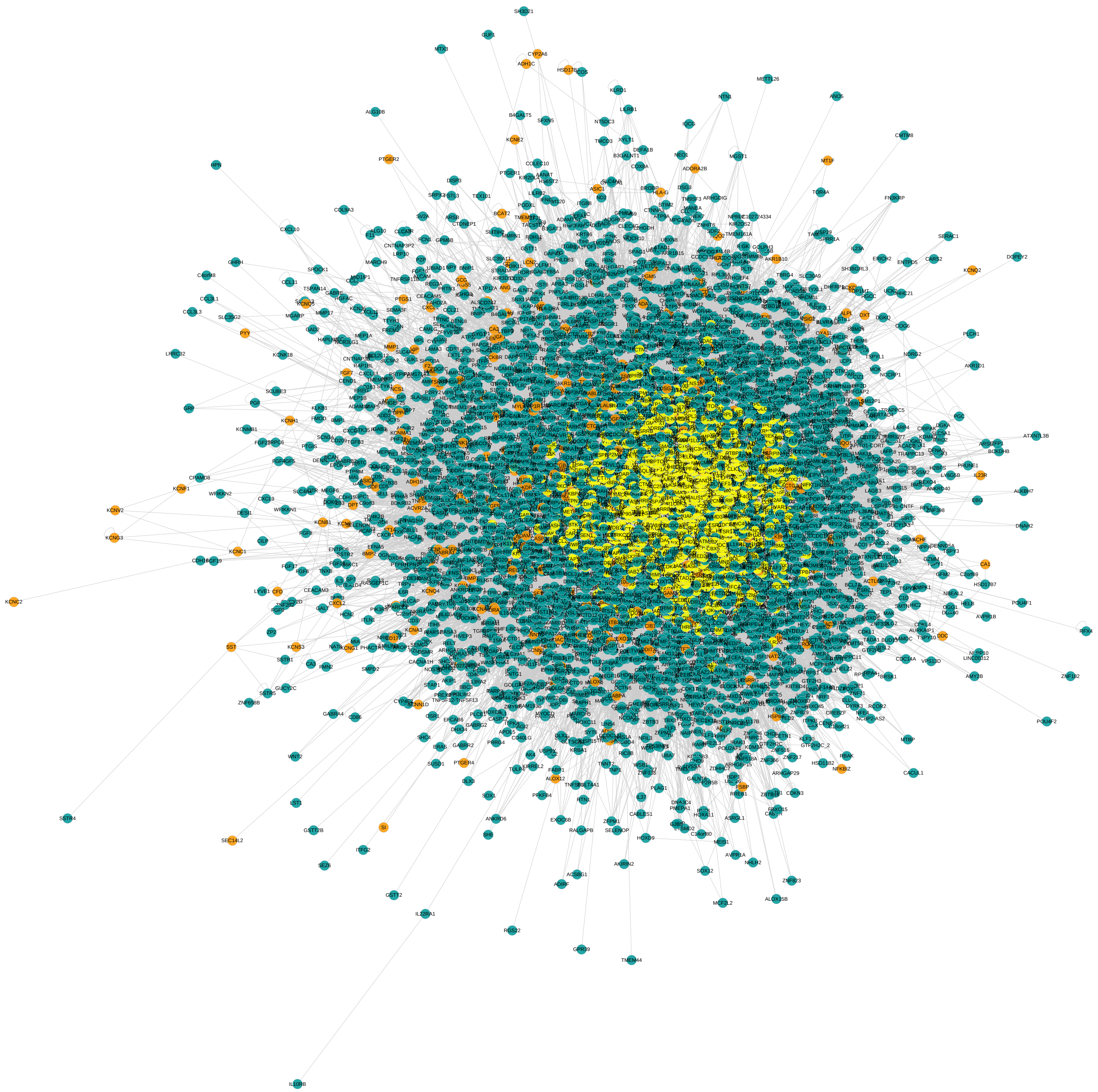

Supplementary Figure 3: Intersection PPI network. 5463 nodes and 147155 edges were identified.

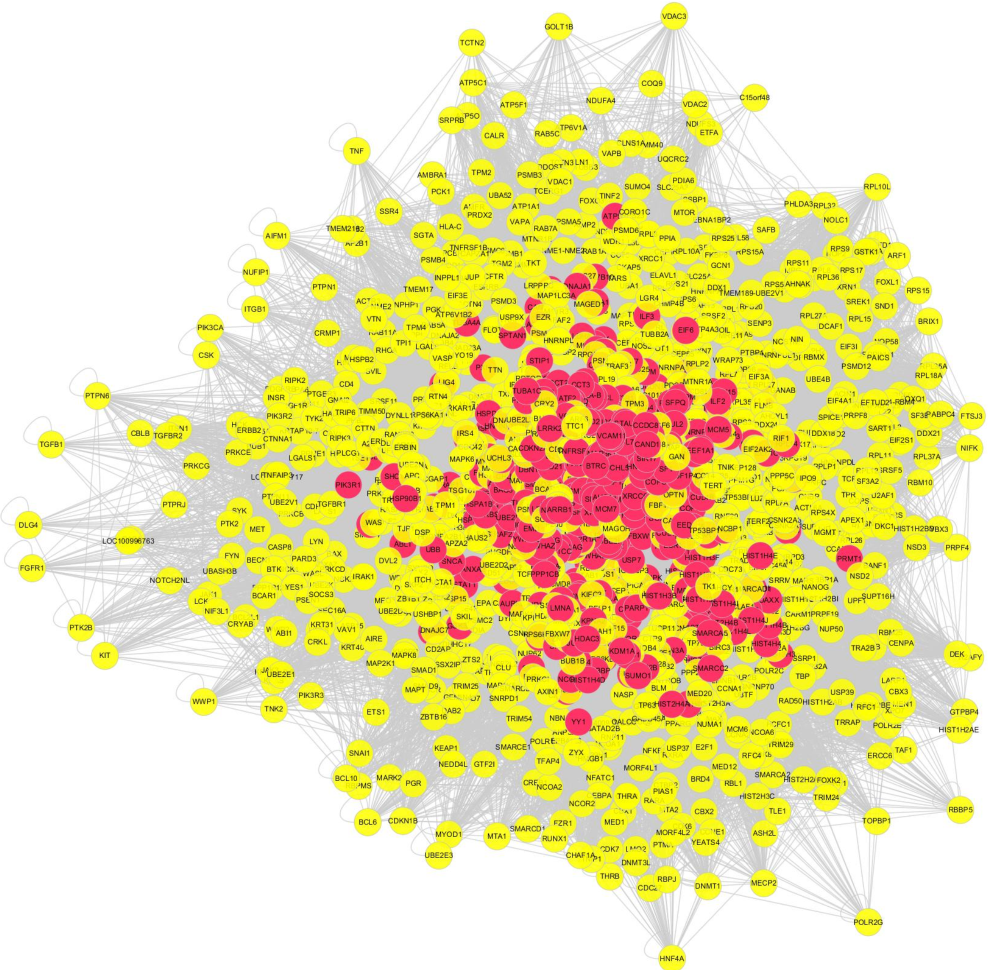

Supplementary Figure 4: Hub PPI network. 1307 nodes and 58,424 edges were identified.
